# The safe and effective use of supercritical CO_2_-processed bone allografts for cervical and lumbar interbody fusion: A retrospective study

**DOI:** 10.3389/fsurg.2023.984028

**Published:** 2023-02-07

**Authors:** Nicolas Aurouer, Patrick Guerin, Arnaud Cogniet, Morad Pedram

**Affiliations:** Centre Aquitain du dos, Clinique du Sport de Bordeaux, 2 rue Georges-Negrevergne, Mérignac, France

**Keywords:** cervical and lumbar fusion, bone allograft, anterior cervical decompression and fusion, anterior lumbar interbody fusion, supercritical CO_2_ treated bone

## Abstract

**Introduction:**

The clinical efficacy and safety of supercritical CO_2_-processed bone allografts prepared from living donors has yet to be confirmed in spinal surgery. Here we report our clinical and surgical experience of using supercritical CO_2_-processed bone allografts for lumbar and cervical fusion.

**Methods:**

Sixteen patients underwent one or two level anterior cervical discectomy and fusion and 37 patients underwent anterior retroperitoneal route lumbar fusion using bone allografts processed using supercritical CO_2_ extraction combined with chemical viral inactivation. Fusion success was assessed radiographically in the immediate postoperative period and at one month, six months, one year, and three years postoperatively. Function and pain were assessed using visual analog scales, Odom's criteria, the neck disability index (NDI), and the Oswestry disability index (ODI).

**Results:**

At a mean of 43 and 47 months postoperatively, 95.3% and 90.5% of cervical and lumbar fusion patients had radiographic evidence of bone fusion, respectively. Over 80% of patients reported good to excellent outcomes according to Odom's criteria, the perception of pain significantly decreased, and the mean NDI and ODI scores significantly improved at the last follow-up compared with before the operations. There were no safety concerns. For the cervical group, the mean NDI score improved from 26.3 ± 6.01 preoperatively to 15.00 ± 8.03 and 17.60 ± 13.95 at immediate post-op (*p* = 0.02) and last follow-up visits (*p* = 0.037) respectively. For the lumbar cases, the mean ODI score improved from 28.31 ± 6.48 preoperatively to 14.68 ± 5.49 (*p* < 0.0001) and 12.54 ± 10.21 (*p* < 00001) at immediate post-op and last follow-up visits respectively.

**Conclusion:**

Within the limitations of this study, the use of supercritical CO_2_-processed bone allografts resulted in satisfactory clinical outcomes and fusion rates with acceptable safety for both cervical and lumbar surgeries.

## Introduction

Cervical or lumbar fusion is a good therapeutic option for a range of degenerative disorders that do not respond to conservative therapy, and spinal arthrodesis is an increasingly common orthopedic procedure (1). Autogenous iliac crest bone grafts (ICBGs) have conventionally been used for cervical or lumbar fusion, as this graft is widely accessible and possesses intrinsic osteoconductive, osteoinductive, and osteogenic qualities that promote osteoblastic proliferation and bone tissue development (2).

However, autogenic ICBGs have significant drawbacks including longer operation times and morbidity related to the need for a second donor surgical site (especially infection, hematoma, fracture, and discomfort) (3–6). To enhance fusion, allografts, graft extensions, and osteobiologics have been used as alternatives to ICBG for spinal fusion. All of these procedures achieve their goals by leveraging biological osteoconductivity, osteoinduction, or osteogenesis (7, 8). Fresh frozen or freeze-dried allogenic bone transplants have some advantages over autogenic bone including reduced surgical morbidity, shorter operating times, and higher availability and quantity (9, 10). Histological and histomorphometric data suggest that allogenic bone possesses equivalent osteoconductivity to autogenic bone (11).

Supercritical CO_2_-processed bone allografts (Supercrit® BIOBank, Lieusaint, France) are synthesized from human femoral heads obtained from living donors during hip replacement surgery. The femoral heads are cleaned and viruses inactivated using a supercritical CO_2_ extraction technique based on delipidation of bone tissue with non-toxic liquid CO_2_ in the supercritical state together with chemical oxidation of remnant proteins contained within the pores of the cancellous tissue (12). The procedure does not influence the mineral and collagen content of the bone matrix, retaining the integrity of trabecular bone tissue and mechanical strength equivalent to fresh bone. As a result, supercritical CO_2_-treated bone is as osteoconductive as autogenic bone (13, 14). The safety of viral inactivation of Supercrit'® has previously been proven (15, 16). Supercrit® has successfully been used in dental surgery for maxillary sinus elevation (17) for extraction socket grafting (18).

While the efficacy of allogeneic bone grafts has been demonstrated for skeletal defect repair, fracture filling, pseudoarthrosis therapy, and spinal fusion in several systematic reviews (19–22), the clinical efficacy of supercritical CO_2_-processed bone allografts has yet to be confirmed in patients undergoing spinal surgery. Here we share our clinical experiences of using Supercrit®-treated bone allografts in patients requiring lumbar or cervical fusion and, in doing so, show that the material is efficacious and safe for this indication.

## Methods

### Patients

This is a retrospective study with no formal sample size calculation performed. From an initial cohort of 60 cases, we reviewed the data of fifty-three (53) patients treated with the BIOBank supercritical CO_2_-processed bone allografts for cervical fusion (*n* = 16 cases representing 21 levels) and lumbar fusion (*n* = 37 cases representing 42 levels) between September 2016 and January 2018, representing approximately 20% of the cases at the institution. Seven patients were lost to follow-up (2 cervical cases and 5 lumbar cases). Enrolment criteria included patients ≥18 years of age with 1or 2-level degenerative disease, with cervicobrachial neuralgia on hernia or disc arthrosis for cervical cases, low back pain or lumbar radicular pain on herniated disc or inflammatory discopathy for lumbar cases. Patients with metastatic tumors or infection were excluded.

### Ethical considerations

This study was conducted in accordance with all applicable regulations and with the principles of the Declaration of Helsinki. Due to it retrospective nature, this study came under the French Data Protection Authority Law (the CNIL) Reference Methodology MR004 for approval and thus did not require formal ethical approval. All patients provided consent before any data collection from their files.

### Graft material

BIOBank cancellous bone allograft granules processed using Supercrit® technology were used as graft material. Allografts were prepared from living donor femoral heads treated with the supercritical CO_2_ process through degreasing steps and gentle chemical oxidation of the residual proteins to preserve the bone architecture. Prior to fusion, bone allograft powder and granules drawn from the cleaned femoral head and packed into a syringe or vial were hydrated with bone marrow blood taken percutaneously with a trocar from the iliac crest.

### Surgical technique

All surgical procedures were performed by five senior orthopedic surgeons. All patients were assessed preoperatively to determine their general health status. Cervical arthrodesis was performed *via* the sternocleidomastoid antero-lateral route (ACDF). Fusion was based on complete discectomy followed by abrasion of the vertebral endplates to viable bone before introduction of an interbody cage in PEEK filled with rehydrated allograft powder complemented with an osteosynthesis plate. Lumbar arthrodesis was performed *via* the anterior retroperitoneal route (ALIF). Fusion was based on complete discectomy followed by abrasion of the vertebral endplates to viable bone and then introduction of an interbody cage in PEEK filled with rehydrated allograft powder associated with small fragments of cancellous bone taken minimally from the iliac crest complemented with an osteosynthesis plate.

### Outcome measures

Postoperative CT scans were reviewed at the last follow-up. The Bridwell fusion grading system was used to classify fusion on a 4-point scale: grade 1: completely remodeled with trabeculae across disc space; grade 2: graft intact with no lucent lines seen between graft and adjacent endplates; grade 3: graft intact, but a radiolucent line seen between the graft and an adjacent endplate; and grade 4: lucency along an entire border of the graft or lucency around a pedicle screw or subsidence of the graft. Based on this classification system, grade 1–2 was regarded as successful fusion and 3–4 as unsuccessful fusion. All patients were evaluated for graft subsidence and migration on the postoperative CT scan at 12 and 36 months.

Clinical outcomes were measured at baseline, at 12 months, and at the last follow-up using four validated health measurement instruments: the neck disability index (NDI) for cervical patients, the Oswestry disability index (ODI) for lumbar patients, the Odom 4-point rating scale for clinical outcomes after spinal surgery (poor, satisfactory, good, excellent) (23). A 100 mm visual analogue scale (VAS; 0 representing no pain and 100 representing severe pain on activity) for neck and arm pain was used for cervical patients and a VAS for lumbar and radicular pain for lumbar patients. The NDI and ODI score up to 50 points, with higher scores representing greater functional improvement.

### Statistical analysis

Statistical analyses were carried out using IBM SPSS Statistics v26 (SPSS Inc. Chicago, IL). Descriptive statistics were analyzed as means (standard deviations, SD) for continuous variables and percentages for categorical variables. Continuous variables were compared using the Wilcoxon-test and categorical variables were analyzed with the chi-squared-test. A *p*-value < 0.05 was regarded as statistically significant.

## Results

### Patient characteristics and fusion rates

**Figure 1:**
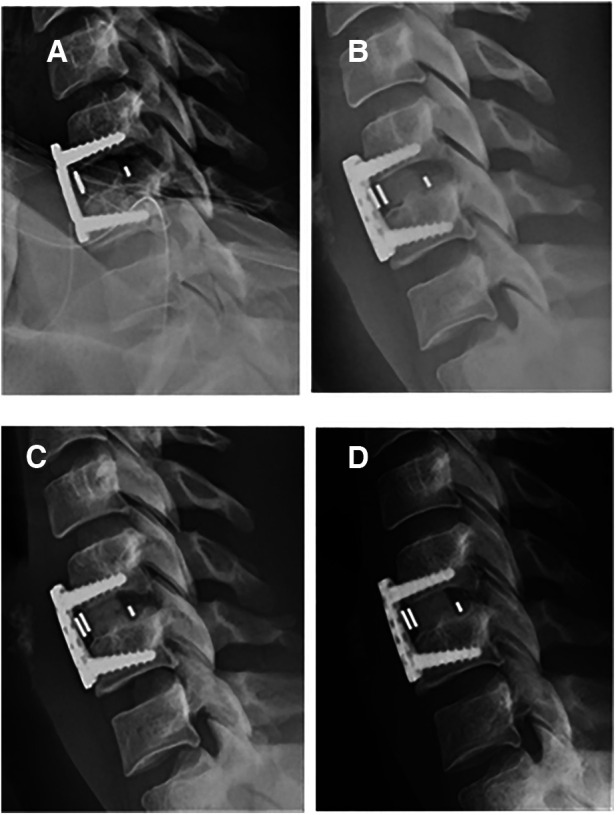
One-level ACDF arthrodesis using BIOBank allograft on a 37-year-old female at C5C6. x-Ray lateral view at immediate post-operative (**A**), 1 month postoperative (**B**), 4.5 months postoperative with visible fusion (**C**) and 1 year postoperative (**D**).

**Figure 2:**
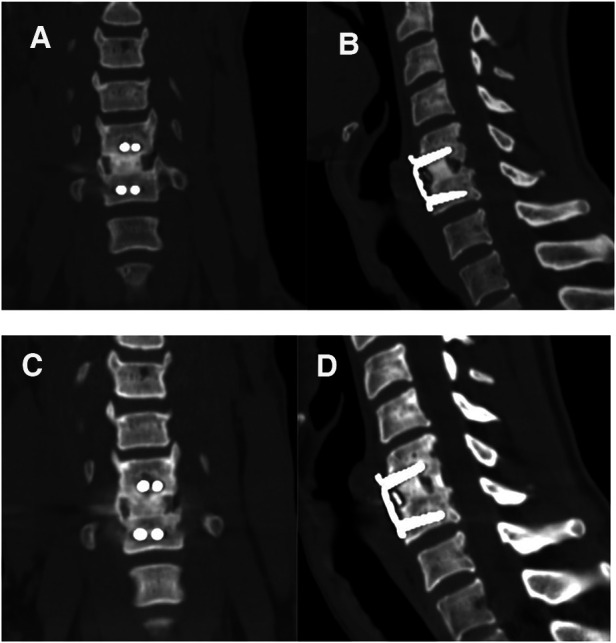
Coronal and sagittal computed tomography scans taken 12 months (**A,B**) and 36 months (**C,D**) after surgery showing satisfactory fusions.

**Figure 3:**
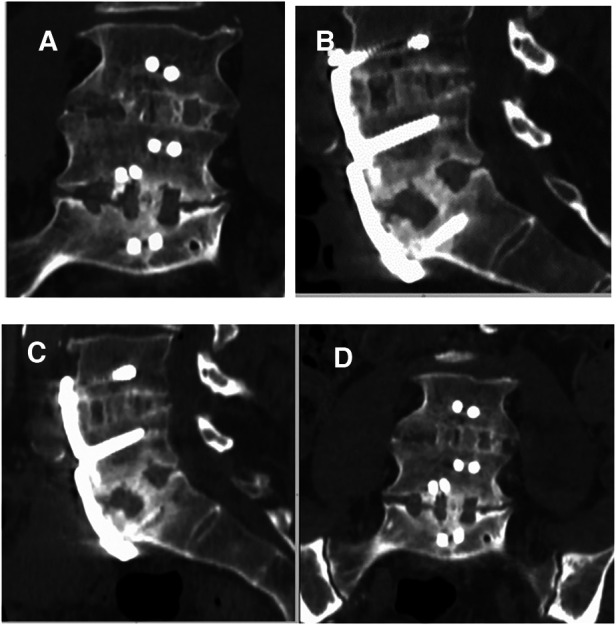
Two-level ALIF using BIOBank allograft on a 58-year-old male at L4L5 et L5S1. Coronal and sagittal computed tomography scans taken 14 months (**A,B**) and 36 months (**C,D**) after surgery showing satisfactory fusions.

At the time of this review, follow-up data was available for 53 patients, 15 cervical and 37 lumbar cases. Their mean age was 49 years (range 33–99) and 48 years (range 32–67) for cervical and lumbar groups respectively. There were 8 females and 8 males in the cervical group and 22 females and 15 males in the lumbar group. The mean follow-up was 43 months for the cervical group and 47 months fir the lumbar group. Examples of successful fusion are shown in Figures 1–3, and the patient demographics, vertebral locations, and fusion rates are detailed in Table 1. The per level fusion rate was 95.3% of cervical cases at 43 months and 90.5% of lumbar cases at 47 months (Table 1).

**Table 1:** Patient characteristics and fusion outcomes.

Variable		Cervical	Lumbar	*P*
Number of patients		16	37	
Gender (%)	Male	8 (50)	15 (40.5)	
	Female	8 (50)	22 (59.5)	0.011
BMI (kg/m²) (mean; range)		24.1 (18.2–29)	24.6 (17.7–36.8)	
Smoking status (%)	Never	12 (75)	24 (65)	
	< 10 packets/year	2 (12.5)	5 (13.5)	
	> 10 packets/year	2 (12.5)	8 (21.5)	
Mean age at surgery (years; range)		49 (33–99)	48 (32–67)	
Operative indications (%)	Foraminal stenosis	15 (71.5)	1 (2.4)	< 0.0001
	Herniated disc	6 (28.5)	2 (4.8)	
	Degenerative disc disease		33 (78.5)	
	Spondylosis		3 (7.1)	
	Pseudoarthrosis		1 (2.4)	
	Revision		1 (2.4)	
	Instability		1 (2.4)	
Number of levels of surgery (%)				
	1-Level	11 (68.7)	29 (78.4)	0.004
	2-Level	5 (31.34)	8 (21.6)	
Fusion location	C3C4	1 (4.8)		
	C4C5	2 (9.5)		
	C5C6	8 (38.1		
	C6C7	7 (33.3)		
	C7T1	3 (14.3)		
	L2L3		1 (2.4)	
	L3L4		3 (7.1)	
	L4L5		14 (33.3)	
	L5S1		24 (57.1)	
Fusion rates (at >42 months)	Successful fusion *n* (%)	20 (95.3)	38 (90.5)	
	Unsuccessful fusion *n* (%)	1(4.7)	4 (9.5)	

### Clinical outcomes

There were significant improvements in all patient-reported outcomes (NDI, ODI, VAS for pain) for function and pain in the immediate postoperative period and at last follow-up compared with baseline (Table 2; all *p* < 0.05). For the cervical group, the mean NDI score improved from 26.3 ± 6.01 preoperatively to 15.00 ± 8.03 and 17.60 ± 13.95 at immediate post-op (*p* = 0.02) and last follow-up visits (*p* = 0.037) respectively. The mean VAS neck and arm pain scores also significantly decreased: The mean neck pain decreased from 5.71 ± 2.52 preoperatively to 2.81 ± 2.30 (*p* = 0.023) and 0.53 ± 0.83 (*p* = 0.09) at immediate post-op and last-follow-up visits while the mean arm pain decreased from 5.60 ± 2.43 preoperatively to 1.25 ± 2.37 (*p* = 0.018) and 0.89 ± 2.26 (*p* = 0.0013) at immediate post-op and last follow-up. For the lumbar cases, the mean ODI score improved from 28.31 ± 6.48 preoperatively to 14.68 ± 5.49 (*p* < 0.0001) and 12.54 ± 10.21 (*p* < 00001) at immediate post-op and last follow-up visits respectively. The mean VAS lumbar pain significantly decreased 6.62 ± 2.51 to 2.30 ± 2.29 (*p* < 0.0001) and 0.36 ± 0.53 (*p* < 0.0001) and the mean VAS radicular pain from 5.70 ± 2.71 to 1.30 ± 2.41(*p* < 0.0001) and 0.19 ± 0.25 (*p* < 0.0001).

**Table 2:** Clinical outcomes from cervical and lumbar fusion.

		Preoperative	Immediate post- operative period	*p*-value (Preop- Immediate post-op)	Last follow-up	*p*-value (Preop – last follow-up)
Cervical						
NDI		26.3 ± 6.01	15.00 ± 8.03	0.020	17.60 ± 13.95	0.037
VAS neck pain		5.71 ± 2.52	2.81 ± 2.30	0,023	0.53 ± 0.83	0.009
VAS arm pain		5.60 ± 2.43	1.25 ± 2.37	0.018	0.89 ± 2.26	0.0013
Odom (% of patients)	Excellent	NA	6.25%		62.5%	<0.06
	Good	NA	87.5%		18.7%	
	Satisfactory	NA	0		0	
	Poor	NA	6.25%		18.7%	
Lumbar						
ODI		28.31 ± 6.48	14.68 ± 5.49	<0.0001	12.54 ± 10.21	<0.0001
VAS lumbar pain		6.62 ± 2.51	2.30 ± 2.29	<0.0001	0.36 ± 0.53	<0.0001
VAS radicular pain		5.70 ± 2.71	1.30 ± 2.41	<0.0001	0.19 ± 0.25	<0.0001
Odom (% of patients)	Excellent	NA	8.1%		43.3%	< 0.08
	Good	NA	83.8%		40.5%	
	Satisfactory	NA	0		0	
	Poor	NA	8.1%		16.2%	

**Figure 4:**
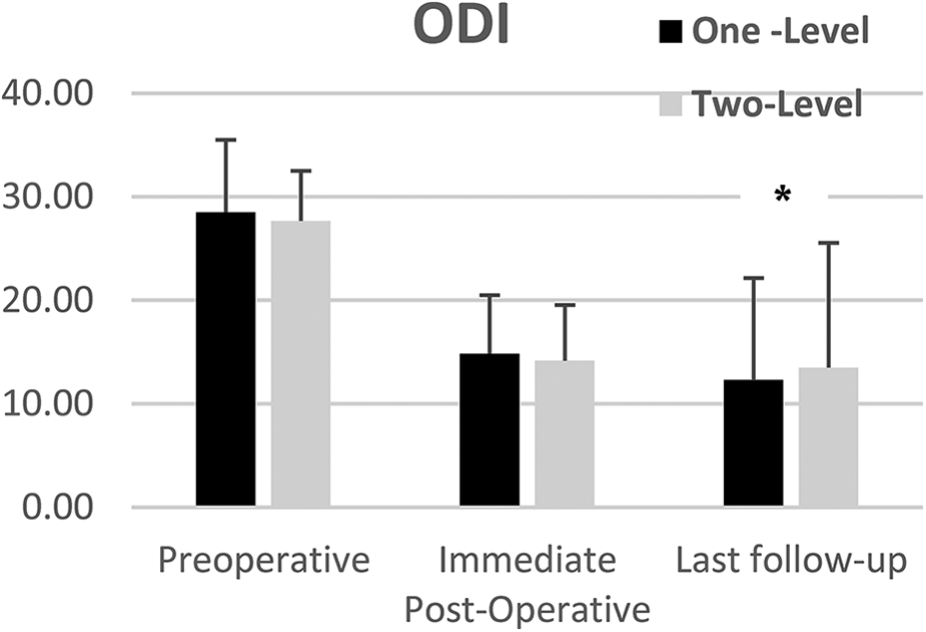
Mean ODI scores of one and two-level cohorts at pre-op, immediate post-op, and last follow-up. One-level and Two-Level groups show statistically significant improvement. Error bars represent standard deviation of the means.

Subgroup analysis showed that ODI scores improved from preoperative to last follow-up for both one-level and two-level treated patients. Mean ODI scores changed from 28.5 and 27.7 preoperatively to 12.3 and 13.5 for One-Level (*p* < 0.001) and two-level patients (*p* < 0.04) respectively with no statistically significant difference between groups (Figure 4) *p* < 0.7.

**Figure 5:**
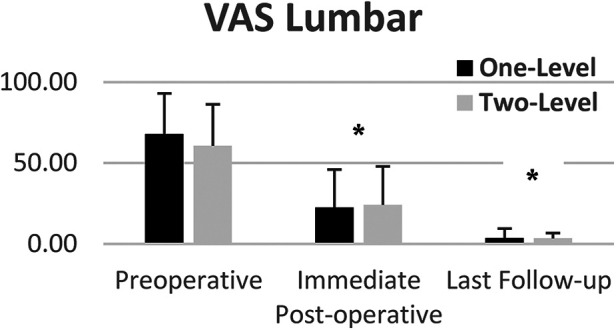
Mean VAS lumbar pain of one and two-level cohorts at pre-op, immediate post-op, and last follow-up. One-level and Two-Level groups show statistically significant decrease in pain. Error bars represent standard deviation of the means.

**Figure 6:**
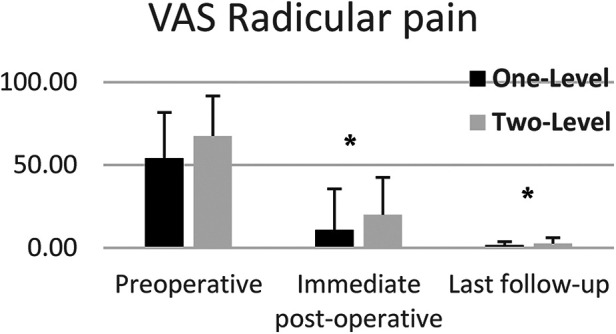
Mean VAS radicular pain of one and two-level cohorts at pre-op, immediate post-op, and last follow-up. One-level and Two-Level groups show statistically significant decrease in pain. Error bars represent standard deviation of the means.

VAS lumbar and radicular pain significantly decreased from preoperative to lats follow-up for both one and two-level subgroups (Figures 5, 6). Mean VAS lumbar pain decreased from 67.8 preoperatively to 3.6 at last follow-up (*p* < 0.0001) for One-Level and from 60.6 preoperatively to 3.5 at last follow-up (*p* < 0.01) for Two-level groups. Mean VAS radicular pain decreased from 54.1 preoperatively to 1.6 at last follow-up (*p* < 0.0001) for One-Level and from 67.5 preoperatively to 2.6 at last follow-up (*p* < 0.01) for Two-level groups. Between groups difference was not statistically significant.

Odom's criteria were excellent in 62.5% of cervical patients at the last follow-up, good in 18.7%, and bad in 18.7%, while they were excellent in 43.3% of the lumbar patients at the last follow-up, good in 40.5%, and bad in 16.2% (Table 2). Subgroup analysis showed improvement in ODOM criteria from 62% good and excellent at immediate postoperative to 86% good and excellent at last follow-up for One-Level treated patients and from 50% good and excellent at immediate post-operative to 75% at last follow-op for Two-Level treated patients. The improvement was not statistically significant between immediate postoperative and last follow-up visits for both groups. The different was not statistically significant between groups *p* < 0.5.

### Safety

No complications were recorded during surgery and at immediate postoperative. There were no adverse events or infections related to the allograft that required revisions. There was no neurological deterioration recorded at any time compared with baseline.

After the study completion, one of the four lumbar cases with unsuccessful fusion reported in (Table 1) who had anterior approach surgery initially, underwent posterior revision surgery for graft complement and additional screwing for consolidation.

One additional lumbar revision surgery occurred to extend the fusion to the upper level and therefore unrelated to the initial fusion.

## Discussion

A variety of biomaterials can be employed for spinal grafting including autografts, allografts, demineralized bone matrix, and/or graft replacements such as ceramic scaffolding devices. To improve fusion rates, a variety of mesenchymal stem cell, growth factor, and synthetic peptide-based approaches have also been tested (24). While ICBG is still the gold standard for cervical and lumbar fusion, it does carry a risk of donor site complications (pain, hematoma, infection) (3–6, 19, 20).

Synthetic bone graft alternatives such as hydroxyapatite (HA) or HA mixed with collagen, tricalcium phosphate, calcium sulfate, or polymethylmethacrylate have been reported to increase the risk of graft fragmentation and settling and have more instrumentation issues when compared with ICBGs (25).

Most commercially-available bone allografts (freeze-dried bone allograft, demineralized freeze-dried bone allograft) are made from cadaverous bone processed in a variety of ways including physical debridement to remove soft tissue, ultrasonic washing to remove remnant cells and blood, and delipidation and viral inactivation with strong organic solvents (12). The bone allografts used in this study were manufactured from the femoral heads of living donors harvested during hip replacement surgery and processed using supercritical CO_2_ extraction, a technique widely used for organic material splitting, extraction, and disinfection in the pharmaceutical and food sectors. The Supercrit® method includes a degreasing stage with supercritical CO_2_ and a moderate chemical oxidation of the remaining proteins in the bone. Preclinical studies have demonstrated that this technique does not influence the composition of bone and retains its architectural and mechanical capabilities, especially its high wettability, thereby preserving performance (13–16).

Allografts have a 93.5% fusion rate when used alone for single- or double-level anterior cervical discectomy and fusion (26) and a 83%–100% fusion rate for lumbar fusion (27). With significant limitations in available literature, systemic reviews conducted in lumbar and cervical spine reported similar effectiveness in terms of fusion rate for allografts compared to ICBG (6, 20). In our study, Supercrit®-processed bone allografts resulted in satisfactory and comparable clinical outcomes, with 95.3% and 90.5% fusion rates for cervical and lumbar surgeries, respectively. Furthermore, for cervical procedures, the allogeneic bone grafts allowed us to avoid autogenic bone graft harvesting altogether, while for the lumbar procedures, use of the allogeneic bone grafts dramatically reduced the volume of iliac crest bone graft required and therefore related morbidity and risks. The use of the material was safe, with reduced surgery time, with no graft site complications nor complications related to the procedure or use of the allograft recorded. Based on this encouraging result, our use of allograft has increased to 50% of our current procedures.

The study has some limitations. It was retrospective with inherent biases. However, we tried to avoid selection bias by doing a wide and careful search and review of patients' records. In addition, the data were recorded prospectively in a standardized manner to reduce any risk of recall bias. The sample size was small due to the single-center nature of the study. The patient population was heterogeneous, some were lost to follow-up, and there was no direct comparative analysis with ICBGs. In addition, the analysis of cervical fusion on CT images was difficult due to the presence of the osteosynthesis material and slices not thin enough.

## Conclusions

The use of supercritical CO_2_-processed bone allografts appears to be a safe strategy for achieving spinal fusion while limiting the morbidity associated with autograft collection. A larger, randomized controlled study comparing allogeneic and autologous grafts is now warranted.

## Data Availability

The raw data supporting the conclusions of this article will be made available by the authors, without undue reservation.
