# Supplementary figures and images for: The safe and effective use of supercritical CO2-processed bone allografts for cervical and lumbar interbody fusion: A retrospective study
Source: Front Surg. 2023 Feb 7;10:984028. doi: 10.3389/fsurg.2023.984028 (PMC9941326; doi:10.3389/fsurg.2023.984028)

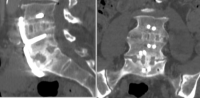

Supplement: Supplementary file 1 [file Image1.png]

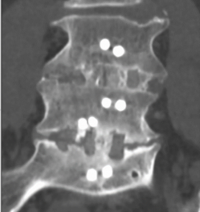

Supplement: Supplementary file 2 [file Image2.png]

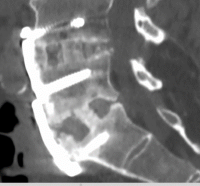

Supplement: Supplementary file 3 [file Image3.png]
